# Burnout and Perception of Medical School Learning Environments Among Gay, Lesbian, and Bisexual Medical Students

**DOI:** 10.1001/jamanetworkopen.2022.9596

**Published:** 2022-04-29

**Authors:** Caitlin R. Ryus, Elizabeth A. Samuels, Ambrose H. Wong, Katherine A. Hill, Stephen Huot, Dowin Boatright

**Affiliations:** 1 Department of Emergency Medicine, Yale School of Medicine, New Haven, Connecticut; 2 Department of Emergency Medicine, Warren Alpert Medical School of Brown University, Providence, Rhode Island; 3 Yale School of Medicine, New Haven, Connecticut; 4 Office of Graduate Medical Education, Yale School of Medicine, New Haven, Connecticut

## Abstract

**Importance:**

The perspectives of gay, lesbian, bisexual (sexual minority [SM]) students about their medical school learning environment and how they relate to burnout is poorly understood.

**Objective:**

To understand SM medical students’ perceptions of the medical school learning environment and how this is associated with reported burnout.

**Design, Setting, and Participants:**

This cross-sectional study included medical students graduating from Association of American Medical Colleges (AAMC)–accredited allopathic US medical schools in 2016 and 2017 and responding to the AAMC Graduation Questionnaire. Data analysis was conducted from June 2021 to March 2022.

**Exposures:**

Sexual orientation, based on self-identification, and categorized as bisexual, gay or lesbian, or heterosexual or straight.

**Main Outcomes and Measures:**

Primary outcomes included burnout as measured by Oldenburg Burnout Inventory for Medical Students (OLBI-MS; two 24-point scales [range, 0-48], with higher scores indicating greater burnout) and student perceptions of the medical school learning environment (0-5–point scales for emotional climate [range, 0-20] and student-faculty interactions [range, 0-15], with higher scores indicating more positive perceptions). Logistic regression was used to model the association between burnout, SM status, and learning environment while controlling for demographic characteristics.

**Results:**

A total of 25 757 respondents (12 527 [48.6%] women; 5347 [20.8%] Asian; 2255 [8.8%] underrepresented in medicine; 15 651 [60.8%] White; 10 726 [41.6%] aged ≤26 years) were included in the analysis: 568 (2.2%) self-identified as bisexual, 854 (3.3%) as gay or lesbian, and 24 335 (94.5%) as heterosexual or straight. Both bisexual students and gay or lesbian students reported less favorable perceptions of their learning environments than heterosexual students (mean [SD] emotional climate score, bisexual students: 8.56 [3.29]; gay or lesbian students: 9.22 [3.33]; heterosexual or straight students: 9.71 [3.20]; *P* < .001; mean [SD] faculty-student interaction score, bisexual students: 13.46 [3.69]; gay or lesbian students: 14.07 [3.45]; heterosexual or straight students: 14.32 [3.37]; *P *< .001). Bisexual and gay or lesbian students were more likely to be in the top quartile for burnout scores (bisexual: odds ratio [OR], 1.71; 95% CI, 1.42-2.07; *P* < .001; gay or lesbian: OR, 1.53; 95% CI, 1.31-1.79; *P* < .001). This association was attenuated when accounting for student perceptions of the learning environment (bisexual: OR, 1.37; 95% CI, 1.11-1.67; *P* < .001; gay or lesbian: OR, 1.42; 95% CI, 1.19-1.68; *P* < .001), with poorer perceptions of the medical school learning environment associated with higher burnout symptoms.

**Conclusions and Relevance:**

In this cross-sectional study, SM students had less favorable perceptions of the medical school learning environment compared with heterosexual students. Results suggest the medical school environment may be associated with higher rates of burnout in SM students. Future research should explore interventions to improve the learning environment for SM students.

## Key Points

**Question:**

Is there an association between perceptions of medical school learning environment and burnout among gay, lesbian, and bisexual (sexual minority [SM]) students?

**Findings:**

In this cross-sectional study of 25 757 graduating medical students, SM students had less favorable perceptions of the medical school learning environment compared with heterosexual students. There was an association between poorer perceptions of medical school and increased levels of burnout.

**Meaning:**

These findings suggest that improving the medical school learning environment and inclusivity of medical school is key in mitigating burnout among SM students.

## Teaser

This cross-sectional study evaluates gay, lesbian, and bisexual medical students’ perceptions of the medical school learning environment and how this is associated with reported burnout.

## Introduction

Diversifying the health care workforce to be more inclusive of physicians who are minoritized on the basis of their sexual orientation (ie, sexual minority [SM] individuals) is an important strategy for addressing health disparities related to sexual orientation.^1,2,3^ Approximately 8% of the US population identifies as lesbian, gay, bisexual, or transgender.^4^ Discrimination against SM persons has been associated with high rates of psychiatric disorders, substance use disorders, and experiences of violence and victimization.^5,6,7,8^ Multiple studies have demonstrated the importance of increasing the diversity of the health care workforce in improving health disparities, patient satisfaction, and financial performance.^9,10,11,12,13^ To that end, several national medical organizations have identified increasing SM representation and development as priorities for addressing health disparities among SM populations.^2,3^ However, a culture of heterosexism and discrimination creates nonwelcoming learning environments, potentially contributing to symptoms of burnout.^14,15,16^ Increased rates of burnout further create a barrier to retention and recruitment of SM health care workers.^14,17,18^

Medical school learning environments are important in fostering students’ professional growth and self-actualization.^19^ These environments may not be equally supportive of all students, as nearly 29% of SM students report not openly expressing their sexual identity, often because of fear of discrimination.^20^ While many factors contribute to students’ perceptions of their learning environment, faculty behavior is influential in establishing cultural norms and climates of inclusion. Faculty modeling of discriminatory behavior is associated with anti–lesbian, gay, bisexual, and transgender bias among students.^21^

Poor perceptions of the medical school learning environment have been shown to be associated higher levels of burnout, lower levels of empathy, and career regret.^22^ Despite the importance of the medical school learning environment, there have been no large-scale studies examining differences in students’ perceptions of learning environments by SM status nor how learning environments are associated with SM student burnout.^14^ To address this knowledge gap, we examined student perceptions of medical school learning environments and self-reports of burnout in a large national cohort of medical students by SM status.

## Methods

### Study Design

This was a cross-sectional study of medical students graduating in 2016 and 2017 who responded to the Association of American Medical Colleges’ Graduation Questionnaire (GQ). The GQ is an annual survey of all graduating medical students from all 140 accredited allopathic medical schools in the United States.^23^ The GQ collects data on student demographic characteristics, career plans, mistreatment, burnout symptoms, and perceptions of the learning environment. The Yale institutional review board deemed this study exempt. This was a secondary analysis of an existing data; the data were deidentified, and informed consent was waived as part of the institutional review board exemption. We conducted study analyses according to the Strengthening the Reporting of Observational Studies in Epidemiology (STROBE) reporting guidelines for cross-sectional studies.^24^

### SM Status

Sexual identity was determined by student self-report and response options included heterosexual or straight, lesbian or gay, and bisexual. Responses were coded as a 3-level variable: gay or lesbian, bisexual, and heterosexual or straight.

### Learning Environment

**Figure 1.**
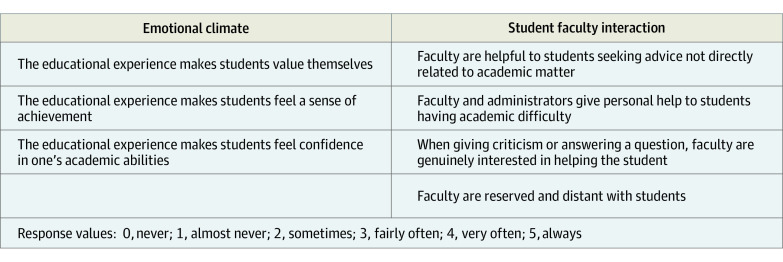
Medical School Learning Environment Survey

The Medical Student Learning Environment Survey (MSLES) is a validated instrument assessing student-faculty interactions and students’ perceptions of the emotional climate (Figure 1). For both components, items are measured on a 0 to 5 point scale. Student-faculty interactions comprise a 4-item component to the instrument and describe perceived distance between faculty and students as well as faculty helpfulness in providing academic advice, nonacademic advice, answering questions, and providing criticism. The emotional climate is a 3-item component that examines students’ sense of achievement, self-valuation, and confidence in academic abilities.^25^ MSLES scores were totaled for each component subscale; 0 to 20 for emotional climate and 0 to 15 for student-faculty interactions. To facilitate comparisons and interpretation, the 2 subscales were transformed into quartiles, with top quartiles corresponding to more favorable environments, consistent with prior research using this instrument.^26^

### Burnout

**Figure 2.**
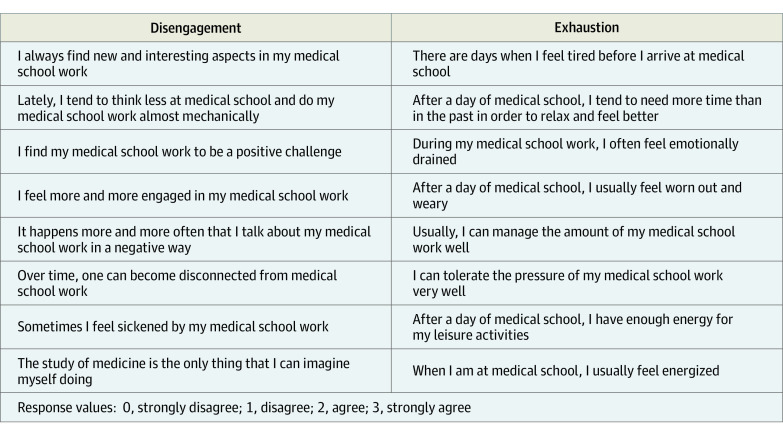
Oldenburg Burnout Inventory–Medical Student

We assessed burnout using the Oldenburg Burnout Inventory for Medical Students (OLBI-MS), a validated instrument that measures 2 dimensions of burnout: exhaustion and disengagement (Figure 2).^27^ The exhaustion subscale explores the cognitive and physical strain from the demands of medical school. The disengagement subscale examines the extent to which students distance themselves from schoolwork and negative attitudes toward medical school. Each dimension is assessed by 8 questions on a 3-point scale, for a total subscale of 0 to 24. Summed scores for each dimension were converted to dichotomous variables to capture the scores in the highest quartile for each subscale to identify students with highest reported burnout symptoms compared with their peers. Additionally, we created a dichotomous variable, top burnout, dividing students into 2 categories: those who scored in the top quartile of both scale dimensions and those who did not, consistent with previous studies using this instrument.^14^

### Inclusion and Exclusion Criteria

We excluded 2767 surveys without self-reported sexual orientation, 3465 with incomplete responses to the OLBI, and 4084 with incomplete responses to the MSLES. In addition, we excluded surveys with missing data for race and ethnicity, sex, age, marital status, school ownership, and student loans. Overall, 4894 surveys were excluded. Race and ethnicity were self-reported. Survey categories were Asian, multi-ethnicity, underrepresented in medicine, White, and other; underrepresented in medicine and other did not specify included racial and ethnic categories. Burnout scores have been shown in previous studies^26,28^ to vary by race and ethnicity and thus were included in our model as covariates.

### Statistical Analysis

We performed descriptive statistics comparing bisexual, gay or lesbian, and heterosexual students’ mean burnout and MSLES scores using analysis of variance. We compared percentages of students in the highest quartile and bottom 3 quartiles of burnout and MSLES by sexual identity using χ^2^ tests. Logistic regression was used to measure the association between burnout, sexual identity, and MSLES scores.

To determine the extent to which perceptions of the medical school learning environment explained the association of identifying as SM with burnout, we first examined the main association by including each of the 3 sexual identity variables (ie, bisexual, gay or lesbian, heterosexual or straight) as factors associated with the burnout outcome. MSLES quartiles were added to the analysis to assess the association between student perceptions of learning environments and burnout symptoms. We then then adjusted for predetermined precision variables that could account for differences in burnout symptoms including sex, marital status, age, race and ethnicity, debt, and private vs public school ownership. Statistical analyses were conducted in 2021 using SPSS version 27 (IBM Corp). All tests were 2-tailed, and *P* < .05 was considered statistically significant.

## Results

A total of 30 651 graduating medical students responded to the survey, a response rate of 80.3%. Of the total 25 757 respondents (12 527 [48.6%] women; 5347 [20.8%] Asian; 2255 [8.8%] underrepresented in medicine; 15 651 [60.8%] White; 10 726 [41.6%] aged ≤26 years) included in our analysis, 568 participants (2.2%) reported identifying as bisexual, and 854 (3.3%) identified as gay or lesbian (Table 1). There were no significant differences in reported sex in the total sample (13 230 [51.4%] identified as male); however, students who identified as bisexual were more likely to be female (400 [70.4%]), and those identifying as gay or lesbian were more likely to be male (663 [77.6%]) (Table 1).

**Table 1.** Demographic Characteristics of Sample

Characteristic	Respondents, No. (%)			
	Total	Bisexual	Gay or lesbian	Heterosexual or straight
Sexual identity	25 757 (100)	568 (2.2)	854 (3.3)	24 335 (94.5)
Sex				
Male	13 230 (51.4)	168 (29.6)	663 (77.6)	12 399 (51.0)
Female	12 527 (48.6)	400 (70.4)	191 (22.4)	11 936 (49.0)
Race/ethnicity^a^				
Asian	5347 (20.8)	98 (17.3)	131 (15.3)	5118 (21.0)
Multi-ethnicity	2259 (8.8)	61 (10.7)	102 (11.9)	2096 (8.6)
URM	2255 (8.8)	52 (9.2)	74 (8.7)	2129 (8.7)
White	15 651 (60.8)	355 (62.5)	545 (63.8)	14 751 (60.6)
Other	245 (1.0)	2 (0.4)	2 (0.2)	241 (1.0)
Age, y				
≤26	10 723 (41.6)	204 (35.9)	328 (38.4)	10 191 (41.9)
27-29	10 635 (41.3)	232 (40.8)	373 (43.7)	10 020 (41.2)
≥30	4409 (17.1)	132 (23.3)	153 (17.9)	4124 (17.0)
Medical loans				
Yes	18 950 (73.6)	430 (75.7)	672 (78.7)	17 848 (73.3)
No	6807 (26.4)	138 (24.3)	182 (21.3)	6487 (26.7)
School ownership				
Private	10 059 (39.1)	258 (45.4)	382 (44.7)	9419 (38.7)
Public	15 698 (60.9)	310 (54.6)	472 (55.3)	14 916 (61.3)
Marital status				
Single	18 877 (73.3)	442 (77.8)	756 (88.5)	17 679 (72.6)
Married or partnered	6585 (25.6)	106 (18.7)	92 (10.8)	6387 (26.3)
Divorced, separated, or widowed	295 (1.1)	20 (3.6)	6 (0.7)	295 (1.1)

Abbreviation: URM underrepresented minority.

^a^ URM and other were answer choices on the original survey that did not further specify additional racial or ethnic categories.

We compared the 4894 students excluded for missing responses with the 25 757 included in the analytic sample to assess bias. A slightly higher proportion of respondents excluded for incomplete OLBI questions identified as a race or ethnicity other than White. Male participants were also slightly less likely to complete the OLBI. There were no differences in SM status or other demographic characteristics between included and excluded respondents.

Both bisexual students and gay or lesbian students reported less favorable perceptions of their learning environments than heterosexual students (mean [SD] emotional climate score, bisexual: 8.56 [3.29]; gay or lesbian: 9.22 [3.33]; heterosexual or straight: 9.71 [3.20]; *P* < .001; mean [SD] score for faculty-student interactions, bisexual: 13.46 [3.69]; gay or lesbian: 14.07 [3.45]; heterosexual or straight: 14.32 [3.37]; *P* < .001). Mean differences were all statistically significant in post hoc *t* tests; however, the largest absolute mean differences were notable between bisexual and heterosexual students. Furthermore, bisexual and gay or lesbian students were less likely to be in the top quartiles (most favorable) of the MSLES than heterosexual students (emotional climate: 140 bisexual students [24.6%]; 277 gay or lesbian students [32.4%]; 8973 heterosexual or straight students [36.5%]; *P* < .001; faculty-student interactions: 102 bisexual students (18.0%); gay or lesbian students 203 [23.8%]; 6160 heterosexual or straight students [25.3%]; *P* < .001) (Table 2).

**Table 2.** Medical Student Learning Environment Survey and Burnout Scores by Sexual Orientation

Outcome	Mean (SD)			*P* value
	Bisexual (n = 568)	Gay or lesbian (n = 854)	Heterosexual (n = 24 335)	
**MSLES**				
Emotional climate				
Total	8.56 (3.29)	9.22 (3.33)	9.71 (3.20)	<.001
The educational experience makes students value themselves	2.77 (1.21)	3.01 (1.24)	3.20 (1.16)	<.001
The educational experience makes students feel a sense of achievement	2.93 (1.15)	3.10 (1.18)	3.29 (1.13)	<.001
The educational experience makes students feel confidence in one’s academic abilities	2.85 (1.12)	3.11 (1.11)	3.23 (1.08)	<.001
Quartile, No. (%)				
Top	140 (24.6)	277 (32.4)	8973 (36.5)	<.001
Second	78 (13.7)	124 (14.5)	3811 (15.6)	
Third	169 (29.8)	225 (26.3)	6472 (26.7)	
Bottom	181 (31.9)	228 (26.7)	5079 (21.3)	
Faculty-student interactions				
Total	13.46 (3.69)	14.07 (3.45)	14.32 (3.37)	<.001
Faculty are helpful to students seeking advice not directly related to academic matter	3.35 (1.20)	3.48 (1.12)	3.53 (1.11)	<.001
Faculty and administrators give personal help to students having academic difficulty	3.29 (1.29)	3.50 (1.21)	3.58 (1.17)	<.001
When giving criticism or answering a question, faculty are genuinely interested in helping the student	3.55 (1.00)	3.68(.98)	3.75 (0.94)	<.001
Faculty are reserved and distant with students (reverse coded)	3.27 (1.10)	3.40(1.01)	3.45 (1.06)	<.001
Quartile, No. (%)				
Top	102 (18.0)	203 (23.8)	6160 (25.3)	<.001
Second	152 (26.8)	233 (27.3)	6791 (27.9)	
Third	165 (29.0)	230 (26.9)	6656 (27.4)	
Bottom	149 (26.2)	188 (22.0)	4728 (19.4)	
**OLBI-MS**				
Exhaustion	12.12 (3.78)	11.55 (3.85)	11.02 (3.6)	<.001
Quartile, No. (%)				
Top	254 (44.7)	326 (38.2)	5449 (22.4)	<.001
Second	123 (21.7)	191 (22.4)	5496 (22.6)	
Third	99 (17.4)	154 (18.0)	5554 (22.8)	
Bottom	92 (16.2)	183 (21.4)	7836 (32.2)	
Disengagement	10.24 (3.86)	10.36 (4.05)	9.74 (3.62)	<.001
Quartile, No. (%)				
Top	196 (34.5)	323 (37.8)	4291 (17.6)	<.001
Second	128 (22.5)	180 (21.1)	7178 (29.5)	
Third	153 (26.9)	209 (24.5)	5623 (23.1)	
Bottom	91 (16.0)	142 (16.6)	7243 (29.8)	

Abbreviations: MSLES, Medical Student Learning Environment Survey; OLBI-MS, Oldenburg Burnout Inventory–Medical Students.

Mean (SD) burnout scores were higher among bisexual and gay or lesbian students than heterosexual students (exhaustion, bisexual: 12.12 [3.78]; gay or lesbian: 11.55 [3.85]; heterosexual or straight: 11.02 [3.60]; *P* < .001; disengagement, bisexual: 10.24 [3.86]; gay or lesbian: 10.36 [4.05]; heterosexual or straight: 20.77 [6.57]; *P* < .001) (Table 2). In our unadjusted model, bisexual and GL students were more likely than heterosexual students to be in the top quartiles for burnout scores (bisexual: odds ratio [OR], 1.71; 95% CI, 1.42-2.07; *P* < .001; gay or lesbian: OR, 1.53; 95% CI, 1.31-1.79; *P* < .001) (Table 3). Notably, in this analysis the association of bisexual and gay or lesbian sexual identities with burnout was attenuated but remained significant after accounting for student perceptions of learning environments (bisexual: OR, 1.37; 95% CI, 1.11-1.67; *P* < .001; gay or lesbian: OR, 1.42; 95% CI, 1.19-1.68; *P* < .001) (Table 3).

**Table 3.** Associations Between Top Burnout Scores and Characteristics of Students

Characteristic	Odds ratios (95% CI)		
	In exhaustion	In disengagement	In both
**Unadjusted model (n = 25 757)**			
Sexual orientation			
Heterosexual or straight	1 [Reference]	1 [Reference]	1 [Reference]
Bisexual	1.70 (1.44-2.01)	1.243 (1.04-1.48)	1.71 (1.42-2.07)
Gay or lesbian	1.33 (1.13-1.50)	1.435 (1.25-1.65)	1.53 (1.31-1.79)
**Adjusted for MSLES (n = 25 757)**			
Sexual orientation			
Heterosexual or straight	1 [Reference]	1 [Reference]	1 [Reference]
Bisexual	1.43 (1.19-1.70)	0.97 (0.80-1.17)	1.37 (1.11-1.67)
Gay or lesbian	1.21 (1.04-1.40)	1.35 (1.16-1.58)	1.42 (1.19-1.68)
MSLES quartile			
Emotional climate			
Quartile			
Top	1 [Reference]	1 [Reference]	1 [Reference]
Second	1.64 (1.50-1.80)	1.27 (1.15-1.40)	1.58 (1.38-1.81)
Third	2.42 (2.23-2.6)	2.21 (2.03-2.41)	2.96 (2.64-3.31)
Bottom	5.50 (5.02-6.03)	4.50 (4.54-5.49)	7.77 (6.90-8.75)
Faculty-student interactions			
Quartile			
Top	1 [Reference]	1 [Reference]	1 [Reference]
Second	1.10 (1.01-1.19)	1.47 (1.34-1.62)	1.17 (1.04-1.33)
Third	1.25 (1.15-1.37)	1.82 (1.65-2.00)	1.38 (1.22-1.56)
Bottom	1.46 (1.32-1.62)	2.74 (2.46-3.05)	1.91 (1.68-2.17)
**Fully adjusted (n = 25 757)** ^a^			
Sexual orientation			
Heterosexual or straight	1 [Reference]	1 [Reference]	1 [Reference]
Bisexual	1.11 (0.92-1.35)	1.32 (1.10-1.58)	1.39 (1.13-1.71)
Gay or lesbian	1.18 (1.01-1.38)	1.32 (1.13-1.54)	1.40 (1.18-1.67)
MSLES quartile			
Emotional climate			
Quartile			
Top	1 [Reference]	1 [Reference]	1 [Reference]
Second	1.65 (1.50-1.80)	1.31 (1.19-1.45)	1.60 (1.40-1.83)
Third	2.44 (2.25-2.65)	2.32 (2.13-2.53)	3.00 (2.68-3.36)
Bottom	5.63 (5.13-6.18)	5.41 (4.92-5.96)	7.95 (7.06-8.96)
Faculty-student interactions			
Quartile			
Top	1 [Reference]	1 [Reference]	1 [Reference]
Second	1.09 (1.00-1.19)	1.48 (1.35-1.62)	1.17 (1.04-1.32)
Third	1.22 (1.12-1.34)	1.80 (1.64-1.99)	1.36 (1.20-1.54)
Bottom	1.41 (1.27-1.56)	2.69 (2.41-3.00)	1.85 (1.62-2.11)

Abbreviation: MSLES, Medical Student Learning Environment Survey.

^a^ Variables adjusted for in the fully adjusted model include sex, race and ethnicity, age, marital status, school ownership, and student loans.

There was a strong association between student reports on the MSLES and burnout symptoms for both faculty interactions and the emotional climate (Table 3). Students reporting decreasingly favorable learning environments were progressively more likely to score in the top quartile of burnout symptoms. Students describing the least favorable compared with students reporting the most favorable faculty interactions were more likely to be in the top burnout quartile (OR, 1.85; 95% CI, 1.62-2.11; *P* < .001). Most strikingly, students in the bottom emotional climate quartile were more likely to rank in the top burnout quartile in the fully adjusted model (OR, 7.95; 95% CI, 6.90-8.96; *P* < .001).

## Discussion

Our study found that the association between SM identification and burnout among medical students was significantly affected by their perceptions of medical school learning environments. After adjusting for the learning environment, the increased likelihood of experiencing top levels of burnout symptoms among SM students decreased from 71% among bisexual students and 53% among gay or lesbian students to about 40% among both groups. SM students reported perceptions of medical schools to be less supportive learning environments than their heterosexual peers, including less helpful faculty-student interactions, and educational experiences that were less likely to provide them with a sense of achievement, value, and academic confidence. Although the mean differences on the MSLES dimensions between SM students and heterosexual students were small, the pronounced overrepresentation of SM students in the lowest MSLES quartiles warrants attention. This disparity in the perceptions of the learning environment is concerning for SM students because students with exceptionally low emotional climate scores were as much as 8 times more likely to be in the highest quartile of burnout symptoms. Furthermore, the association of less supportive medical school environments with burnout followed a stepwise trend: as faculty interactions and emotional climate quartiles became less favorable, the ORs of burnout symptoms increased. The results of our study highlight the medical school learning environment as a vital component of burnout among SM students.

Another notable finding from our analysis was that students who identified as bisexual had higher burnout and lower MSLES scores than both heterosexual or straight students and gay or lesbian students. The largest mean differences in MSLES and OLBI scores were between bisexual and heterosexual or straight students. Among students identifying as bisexual, nearly 40% were in the bottom quartile for emotional climate compared with 21% percent of students identifying as straight. This finding is unsurprising given a body of literature showing a greater disproportion of poor mental health, suicidality, poor physical health, and barriers to health care among people identifying as bisexual compared with those who identify as monosexual.^29,30,31,32,33,34^ These negative health outcomes have been theorized to be attributable to discrimination, biphobia, and erasure.^29,35^ Prior research into medical education and SM students has aggregated SM identities, possibly obscuring differences between sexual identities.^14,15,36^ Further research is need to examine disparate burnout and learning environment outcomes across the range of sexual identities.

The association between burnout among SM students and the medical school learning environment is of critical importance. The undergraduate medical school culture is an opportunity for early intervention in the career trajectories of SM students and subsequent diversification of the physician workforce. Experience of adversity during undergraduate medical education has been shown to significantly affect specialty selection.^37^ A recent study showed that 67% of medical students minoritized on the basis of sexual orientation or gender identity (sexual and gender minority [SGM] students) had concerns that the disclosure of their identity would negatively affect their future career, and nearly 40% of these students had been explicitly advised to avoid disclosing their identity.^36^ Therefore, elucidating the elements of the medical school environment that are most attributable to burnout can help to inform interventions that will be effective and appropriate. Mistreatment has been established to be experienced at increased rates among SGM students and associated with burnout.^14,15,36^ Addressing mistreatment of medical students is a vital component of addressing burnout in this student population.

To foster inclusive learning environments, medical schools must make conscientious efforts to address climate and faculty relationships. The survey used in this study was administered among graduates. However, medical schools would be well served by investigating student experiences in real time, prior to graduation. Surveys administered by confidential third parties at each year of training could help institutions assess their students’ needs and measure the efficacy of interventions implemented while protecting the identities of vulnerable students. It is imperative that such real-time inquiries are followed up with meaningful responses to the results. Prior work has shown that SGM trainees and practitioners reported that having role models with similar identities was valuable when choosing specialties.^37^ Faculty-student interactions could be improved by increasing access to SGM faculty that choose to openly disclose. Active recruitment of SGM individuals at all levels of training should thus be pursued by academic medical institutions to meet this need.

### Limitations

This study has several important limitations requiring discussion. Fear of being identified as an SM individual may result in underreporting of SM status on the survey. Identifying as SM on the GQ does not indicate that students openly disclosed their SM status throughout their training. Thus, we cannot say how much of the learning environment scores with associated with overt discrimination.^38^ This study was further limited by a lack of data on respondent gender identity and limited survey options for SM excluding other common identifiers (such as queer, asexual, pansexual), also resulting in underreporting of identities. Additionally, while our model adjusted for other demographic variables, our analysis does not account for intersectionality. The intersection of SM identity and other identities as it relates to burnout was beyond the scope of this study but is an important area of future research.

## Conclusions

In this study, SM students had less favorable perceptions of the medical school learning environment and were more likely to experience burnout than their heterosexual peers. Future research is needed to identify specific elements of learning environments that are most associated with burnout among SM students. Addressing the experience of medical school learning environments among SM students could improve the inclusivity of medical schools, mitigate burnout, decrease attrition, and ultimately diversify the health care workforce.
